# A Didactic Escape Game for Emergency Medicine Aimed at Learning to Work as a Team and Making Diagnoses: Methodology for Game Development

**DOI:** 10.2196/27291

**Published:** 2021-08-31

**Authors:** Laure Abensur Vuillaume, Garry Laudren, Alexandre Bosio, Pauline Thévenot, Thierry Pelaccia, Anthony Chauvin

**Affiliations:** 1 Emergency Department CHR Metz-Thionville Metz France; 2 Intensive Care and Anesthesiology Pediatric Necker Hospital Paris France; 3 Emergency Department Hospital Center of Verdun Verdun France; 4 Université de Lorraine Nancy France; 5 University of Strasbourg Medical School Strasbourg France; 6 Prehospital Emergency Care Service (SAMU 67) Center for Training and Research in Health Sciences Education Strasbourg University Hospital Strasbourg France; 7 Emergency Department, Lariboisière Hospital Assistance Publique-Hôpitaux de Paris Paris France

**Keywords:** training techniques, educational technique, game theories, emergency medicine, games, education, escape game, simulation-based training, pedagogical, serious games, emergency medicine training

## Abstract

**Background:**

In the health care environment, teamwork is paramount, especially when referring to patient safety. We are interested in recent and innovative solutions such as escape games, which is a type of adventure game that may be highly useful as an educational tool, potentially combining good communication skills with successful gamification. They involve teams of 5 to 10 individuals who are “locked” in the same room and must collaborate to solve puzzles while under pressure from a timer.

**Objective:**

The purpose of this paper was to describe the steps involved in creating and implementing an educational escape game. This tool can then be put into service or further developed by trainers who wish to use it for learning interprofessional collaboration. Therefore, we started with an experience of creating an educational escape game for emergency medicine teams.

**Methods:**

We chose to develop an educational escape game by using 6 successive steps. First, we built a team. Second, we chose the pedagogical objectives. Third, we gamified (switched from objectives to scenario). Next, we found the human and material resources needed. Thereafter, we designed briefing and debriefing. Lastly, we tested the game.

**Results:**

By following these 6 steps, we created the first ambulant educational escape game that teaches people, or nurses, doctors, and paramedics, working in emergency medicine to work as a team.

**Conclusions:**

From a pedagogic point of view, this game may be a good tool for helping people in multidisciplinary fields (medical and paramedical teams) to learn how to work collaboratively and to communicate as a group. Above all, it seems to be an innovative tool that complements medical simulation–based learning and thus consolidates traditional education.

## Introduction

In France, emergency medicine, especially as it pertains to prehospital settings, involves a team of complementary practitioners, including doctors, nurses, ambulance drivers, and nurses’ assistants, who must all work as a team. For this team, 2 aspects are essential: communication and time management. Above all, communication in teamwork is the foundation of effective medical care and is associated with a significant reduction in adverse events [1,2]. Communication within the team is essential, but it is poorly taught, including during continuing medical education [3]. Sharara-Chamia et al [3] reported that emergency physicians lack knowledge about communicating in teams. Currently, there is no consensus on how to effectively teach these skills in health education [4]. To improve teamwork communication training, several educational tools, including medical simulation-based tools, have been developed [3,5].

Gamification is a technique rooted in active learning approaches and is currently being developed, especially in health education [6], and it could satisfy this need. This technique involves applying game design elements to traditionally nongame contexts, such as conventional learning activities [7] and is an innovative tool [8]. According to Rutledge et al [7], gamification differs from serious games, in that it is applied to an existing learning activity or curriculum. Its aim is to help students to meet the objectives of the activity or curriculum. This technique increases participant motivation and engagement [8], which are major determinants of learning [9]. Gamification has a major impact on motivation [7,10,11]. It complements both resident subspecialty and undergraduate student education and can be integrated into simulation-based training [12].

We were interested in recent and innovative solutions such as escape games, which is a type of adventure game that may be highly useful as an educational tool and could combine learning good communication skills with successful gamification. It involves teams of 5 to 10 individuals who are “locked” in the same room and must collaborate to solve puzzles while being subject to a time limit. The main goals of escape games are to develop teamwork and promote concepts used for crew resource management [13-15]. Currently, most escape rooms are supported by private start-ups and are exclusively aimed at the leisure market. The game takes place in the presence of a facilitator, who may be with the players or outside the room (usually with video surveillance and possible ongoing communication with the players). These games are often used to promote team-building in companies. These games are already used in a relevant way in school education [16] and for specific knowledge-learning objectives for pharmacists, for example [17]. Therefore, it may be of interest to use this new tool for training medical teams in interprofessional cooperation [18]. We believe that this tool can effectively teach interprofessional cooperation, especially communication, which is needed to efficiently delegate tasks and use the available time [18,19] to meet the demands of serious, real-life clinical situations. Additionally, the resolution of 1 scenario suggests a collegial decision, which could be placed in the context of medical uncertainty [20].

While the scientific literature on educational escape games and how to evaluate their educational impact is growing [18], point-by-point methods on how to create such games in health education have been poorly described. The purpose of this paper is to describe the steps involved in creating and implementing an educational escape game. This tool can then be put into service and further developed by trainers who wish to use it for learning interprofessional collaboration. Therefore, we started with an experience of creating an educational escape game for emergency medicine teams.

## Methods

### Methods Overview

An educational pilot game was conducted to apply 6 steps of developing escape games to the emergency medicine field. Therefore, a multidisciplinary team was set up, which comprised 5 experts in gaming and gamification. The escape game was created between November 2018 and May 2019 through monthly meetings. Our escape game scenario and all its game components have been submitted to the French National Institute of Intellectual Property for commercial protection. An evaluation is underway and will be the subject of another paper.

To develop an educational escape game, we proposed the implementation of 6 successive steps, which are summarized in Table 1.

**Table 1:** The 6 steps of developing an escape game.

#	Step	Objectives
1	Build a team	To create 2 or more subgroups: 1 that worked on the design of the game and 1 that worked on the educational/scientific content
2	Choose educational objectives	To create a synopsis of the escape game To choose 2 levels of educational objectives: those that are common to all team members and those that are specific to team members’ professions.
3	Gamify: switching from objectives to scenario	To combine the educational objectives of gameplay with its interactive elements and thus develop the educational content of the game
4	Find human and material resources	To provide a facilitator role To list the necessary material To assemble and create the game
5	Create the briefing and debriefing phases	To write a rule book To write an introduction/briefing of the game To write the key steps to guide debriefing
6	Test the game	To test the game and material with real teams and to ensure that the diagnostic process can be followed in the allotted time. To identify key points in the game where players might need help. To write a guide for the facilitator To evaluate the educational impact using conventional scales

### Step 1: Build a Team

In our experience and opinion, the team needed to be composed of 2 to 5 people. In our opinion, all team members must always have had considerable experience as players or in designing game scenarios, including escape, role play, live-action role play, or board games. We believe it is important to have an experience as a player to create this type of tool to better advice development through real-life knowledge. The second point, to which we draw attention, was that it was essential that the professional’s skills of team members be adapted to the target audience. The creation of subgroups makes it possible to work more efficiently by harnessing the specific skills of each group member.

### Step 2: Choose Educational Objectives

To develop the game, it was possible to start from a synopsis (global idea of a scenario in which a clinical case will be inserted) or to start from a clinical case and imagine a synopsis. In fact, the synopsis can be derived from the objectives, or the construction of the synopsis involves defining learning objectives.

It was important to choose 2 levels of educational objectives: those that are common to all team members and those that are specific to each team member’s profession. The common objectives toward learning communication skills in teamwork were (1) expressing ideas, knowledge, and opinions clearly, concisely, and kindly; (2) acquiring knowledge about the professional skills of the other team members; and (3) sharing responsibility for the result in a collegial fashion.

The medical and paramedical objectives represented clinical situations presented in the game. Its level of complexity and content must be adapted in accordance with the skills of the participants. This content must comply with current recommendations and consensus. This scenario allowed participants to generate the most probable diagnostic hypotheses regarding the symptoms and then to restrict the scope of the hypotheses by collecting and interpreting additional data to best fit the expert’s reasoning [21].

### Step 3: Gamify—Switching From Objectives to Scenario

This step combined the educational objectives of gameplay with its interactional elements, thus forming the educational content of the game. For such a game to be successful, the players needed to have a gaming attitude [22]. This playful attitude was defined by Henriot [22], who stated that to play, one must enter the game and play a role in it. In simulation-based training, where participants assume their own or a given role, the escape game requires that the players must adopt a player’s attitude to learn lessons from the game. We can thus observe the group being created and see a natural distribution of tasks (reading, searching, and puzzles being solved in accordance with individuals’ skills), often with the emergence of a leader.

In the same spirit of play, the scenario did not necessarily have to reflect an actual situation but it should have been realistic enough so that learning can be readily transferable to actual clinical situations, consistent with the “exposition” element of Nicholson’s RECIPE for meaningful gamification [23]. However, the scenario was fictitious. The choice of the setting (contemporary, futuristic, or uchronic era) and history was left to the creators. The setting was important because it created the dimension of “play” and placed the learners in a gambling situation.

As in simulation-based training, the escape game was based on reality. Although the setting may be modified, the clinical scenario chosen must be authentic. To create the scenario, the group must think about all of the probable decision-making paths the players could take, allowing the players freedom in the game. This scenario was the decisional algorithm of the game, providing the different paths that could be followed by the learners.

It was important to think about the creation of the game beforehand so that the players can see it to completion. The success of the game lay in allowing it to fully unfold so that the players could have all the elements to make their collegial decision (relevant or not). The important factor was not the outcome but rather the way in which it was achieved.

Gameplay interactions could be chosen in accordance with player strengths, experiences with escape games, or specific support research (eg, the “now escape” website [24]). To ensure that the diagnostic process could be completed in the allotted time and that the players experience adequate time pressure, the game should include 3 to 4 gameplay interactions—or play elements—that require handling (eg, codes, locks, or puzzles) over 10 minutes of gameplay.

As the game progresses, the in-game content makes it possible to suggest all the probable etiologies of the fixed diagnostic setting and to eliminate the various hypotheses. The elements that lead to the right diagnosis are only provided after all components have been presented. The game’s progress was thus essentially controlled by the timepoint configuration of the game elements. Upon conclusion, players must have had all cards in hand. They could then make their final and collective decision and be able to explain their reasoning.

### Step 4: Find Human and Material Resources

#### Integration and Roles of a Facilitator

The facilitator had several roles: (1) to introduce the game: the details of the game can be presented by various supportive elements such as a video or cards; (2) to guide the game and bring in new elements if necessary (eg, changes in the clinical situation or data that would be sought by participants as part of their diagnostic process, but are not available) with strict time control; and (3) to control the game duration by assisting if necessary. The facilitator must be familiar with the entire course of the game and all possible paths or outcomes.

The facilitator could be located with the players or outside the room with voice access and video surveillance. To make the game more immersive, we proposed to position the facilitator directly with the players and thus give him/her a key role with the players. This made it easier to guide the players during the game. In addition, this approach allows for fewer technical and financial constraints. However, to ensure full player immersion in the game, the facilitator must be completely committed to the game and may require actor training. He/she must fit perfectly into the scenario and have a valid role in the story.

#### Listing the Material

The set of material required for the game should be listed at the same time during scenario creation. It should include (1) the clinical case and background elements; (2) padlocks of different types (code, key, etc) that can be fixed to boxes, books, etc; and (3) more elaborate puzzles (labyrinths, sets of magnets, etc).

To represent the etiological elements of the clinical case, each diagnostic etiological component could be represented as an object or symbol in the game (eg, a letter). Set elements were important because they place the learners in the game situation and make it more immersive, but this also allows hiding of clues.

#### Assembling the Material

To promote a hypothetical deductive diagnostic approach (as we mentioned earlier), the material must be presented in a precise way and the order of opening the padlocks must also be specified. Hence, for example, to open box B, a clue needed to be present in box A. In addition to the role of the facilitator, these elements allowed the order of discovery of the diagnostic guidance elements to be controlled. The order of these elements must remain within a clinical logic, as in the simulation.

### Step 5: Create the Briefing and Debriefing Phases

As in simulation-based training, 2 essential phases of the escape game, namely briefing and debriefing, must be carried out.

#### Briefing

Briefing was intended to allow the players to know the questions they have to answer. It should also make the game immersive. This was best achieved by the facilitator and his/her acting skills during the introduction of the game. However, before entering the playground, details of the mission could be presented by various supportive elements (eg, video or cards), but must be adapted to the context of the synopsis.

#### Debriefing

Debriefing was a key step because it reinforces the learning process [25]. As in aeronautical debriefings, the investment of the facilitator in the game is inversely proportional to the level of investment of the group being trained [26].

It is essential that the facilitator adapts his/her debriefing to the level of the group. Each debriefing was unique in its content but has been structured on the basis of written guidelines before the game starts.

While the structure of the debriefing was standardized, the content was not. The standardized structure covers all the main components of scientific content and links them with the game’s progress. By contrast, communication of the team was specific to each group.

### Step 6: Testing of the Game

#### Initial Testing for Adjustments and Creation of the Animation

This type of test allowed us to see if the puzzle sequence was the right one. It was designed not to evaluate the game but rather to highlight any possible inconsistencies in the game’s sequence.

After purchasing the materials for the game (chosen in accordance with the elements described above) and setting up all the play mechanisms, we recommended that the game be tested with “naïve” players; that is, players who were not part of the game development process.

This test was very important because it allowed for refinement of both the game and the game documents and helped correct elements that do not unfold as planned. This test also makes it possible to observe the timing of the game in real life and how facilitating certain elements of the game can improve it. In addition, the test reveals the key timing points of the game, which must occur to allow the game to progress and end in the allotted time. These key points must be completed at predetermined points in the game. The test can also highlight facilitating elements that the facilitator could use to ensure that the game is completed on time. For example, depending on how the scenario develops, the facilitator could communicate with the group providing planned elements and sample sentences that contain clues.

The final step was to document the entire game algorithm along with all of its facilitating and game elements. It was also necessary to create debriefing sheets to help the facilitator complete his/her role. The game must be standardized and follow a specific protocol in terms of how it was presented and when its elements are brought into play. This protocol was written for and presented to the facilitator. The facilitator must be trained specifically on how the game should progress, his/her attitude to the game, and the nature of the facilitating elements.

#### Assessment of Educational Impact

This evaluation phase was important because it allowed the game to evolve further. There are several ways to evaluate an educational model. Of these, the most widely used was Kirkpatrick model [27] with 1 to 3 levels. It was possible to carry out an evaluation in the following way: obtaining participants’ satisfaction with a focus group (level 1), partially because it was acquired from participants in a declarative manner with a remote questionnaire (level 2), and determining perceptible changes in practice in a declarative way using a remote questionnaire (level 3) [27]. We believe that an educational escape game allows reaching the first 2 levels of this scale. This evaluation of educational impact should be carried out systematically for each new health training course and would allow for improvements in gameplay as the game progresses.

## Results

### Step 1: Build a Team

We created a team of 4 people who all had in common a wealth of experience as players and creators of gaming content. The group was coordinated by 1 of the 2 emergency physicians who also had experience in medical education. The game design group consisted of a game designer/nurse anesthetist and a player (non–health professional). The educational/scientific group consisted of 2 emergency medicine physicians. These subgroups worked both separately and collaboratively.

### Step 2: Choose Educational Objectives

Our game was aimed at medical residents, junior physicians, nurses and assistant nurses, and paramedics who work in the emergency department and who require initial and continuing education.

We chose to start from the synopsis and then worked toward the educational objectives. A synopsis is the overall concept of the game. The synopsis emerged after brainstorming (Textbox 1) and allowed us to develop all the objectives.

**Synopsis.**

“An attack occurred on an airliner. The terrorist is gravely ill and about twenty passengers are showing suspicious symptoms. You are a team of specialists commissioned by the local authorities as part of an emergency plan to solve this problem. You have 30 minutes to report before the information is released to the general public. Final diagnosis:

The suspected terrorist has severe pulmonary embolism, and his clinical condition is aggravated by poisoning with nitric oxide”

Our pedagogical objectives were to develop communication within the team and to accompany the learners in a diagnostic reasoning within a complex situation.

Our group objectives were as follows: (1) analyzing the diagnostic process at the medical and paramedical level; (2) analyzing the elements reported by third parties in a clinical situation (eg, firefighters or family members); (3) building a sense of observation; and (4) developing curiosity.

Our specific objectives (including medical and paramedical ones) were as follows: to examine the etiology of nontraumatic coma, to examine the etiology of pulmonary embolism and other causes of acute respiratory failure and make a diagnostic process of pulmonary embolism, observe clinical presentations of nitric oxide poisoning, and participate in a disaster medicine environment with multiple victims.

Our players had to solve a complex clinical case within a multi-victim situation. In our game, the learners played the role of an emergency medicine team: specific roles were not assigned to retain the gaming spirit. The roles would have to be quickly assigned, either spontaneously or allocated by a leader. Then, by reading the information contained in the “victim files” and by searching the suspected terrorist’s belongings, the team members can understand their current state of health. Furthermore, external events may disrupt the game.

### Step 3: Gamify—Switching From Objectives to Scenario

**Figure 1:**
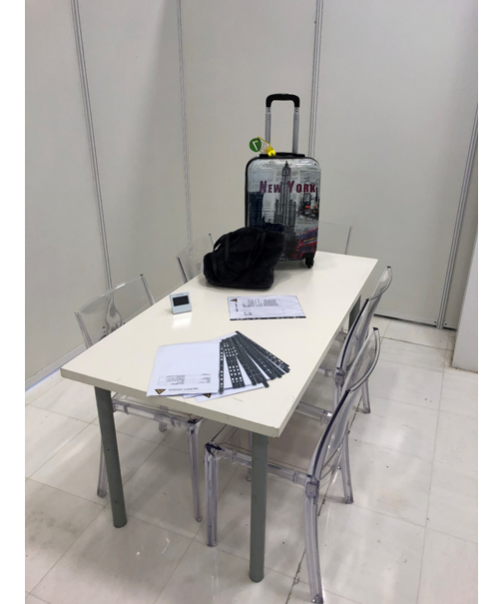
Contents of the escape room that was used for the emergency medicine game in this study.

From our synopsis and objectives, we then developed a more detailed scenario. The team of disaster medicine specialists is dispatched on an emergency plan in the context of a highly probable bioterrorist exposure with approximately 20 victims. An introductory video is shown in which the local authorities explains the situation. A suspected terrorist is currently in a very serious condition and has exposed 20 passengers to an unknown substance. The team has 30 minutes to solve the problem because the local authorities must report to the president of the French Republic within this time limit. For our game, the initial clinical case was presented in hard copy at the end of the video. This hard copy fit into the scenario as a medical document that was created and was not a representation of the patient (Figure 1).

In reality, the young, female terrorist has a real medical problem. She presents with a serious pulmonary embolism, and this is aggravated by group poisoning with nitric oxide. The players have the following at their disposal: the passengers’ medical files and the medical file of the terrorist, as well as her personal belongings, to solve the problem. The players have to solve a complex medical problem: that of the terrorist and of the other victims. In addition, they have to conclude through logic deduction (or not) on the risk to the local population, with respect to this attack.

**Figure 2:**
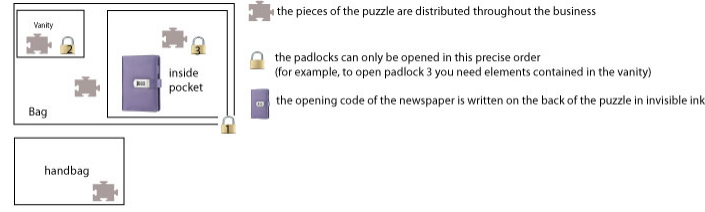
Diary enigma.

Our 30-minute game included 8 gameplay interactions in the form of a puzzle or handled objects. We chose the gameplay interactions on the basis of our preferences as players, our experiences with escape games, and our research on the “now escape” website [24], which contains valuable ideas for gameplay interactions. For example, the final element of the game was a young woman’s diary, which explained her background and allowed for an understanding of the entire clinical case and to deduce the clinical diagnosis. This puzzle is detailed in Textbox 2; we need to see this nesting like a Russian doll (Figure 2).

**Diary puzzle.**

The final element of the game was the young woman's diary, which explained her entire background and allowed for an understanding of the entire clinical case and also to deduce the medical diagnosis. This diary was locked with a 4-digit code. The code was hidden in invisible ink on the back of a puzzle that the players had to reconstruct. (1) the blue light lamp was hidden in the young woman's handbag and (2) the pieces of the puzzle were distributed throughout the young woman's belongings. Once the puzzle was reconstructed and turned over, the code could be found. However, in order to obtain all the pieces of the puzzle, it was necessary to succeed in opening all the locks (suitcase, vanity, second part of suitcase, etc).

The facilitator served as a link between the doctor taking care of the patient and the victims (physically separated from the group so as to not “contaminate the team”) and the expert team. In addition, the facilitator could intervene to guide the players and provide them with additional medical evidence but also the progress of the terrorist’s and passengers’ symptoms, to guide the players or to provide them with additional medical documents (a laboratory result, an electrocardiograph, or an X-ray). To make the game mobile, we chose to give players access to the patient’s personal suitcase and bags in a closed room. The players were in this room with the facilitator.

### Step 4: Find Human and Material Resources

The facilitator was trained in how to conduct the game and the role he/she was expected to play.

Other clues pointed to other diagnoses that needed to be excluded as the game progressed Similarly, we applied this approach to the group diagnosis, which could be elicited through careful clinical analysis. All the materials were listed by the group (an example of the material for the diagnosis of pulmonary embolism is provided in Textbox 3).

**Game elements and material for the diagnosis of a pulmonary embolism: an example.**

Pulmonary embolism was the probable diagnosis because of the following reasons [28]:

- *Elements of the story: long-haul plane flight*
- *The young woman's belongings included, a cigarette packet, birth control pills, compression stockings, and a photo of her in a cast*
- *Clinical presentation: acute respiratory failure, an electrocardiogram and a typical blood gas picture. (communicated by the facilitator at H+25mn)*
- *A positive pregnancy test and positive d-dimer (communicated by the facilitator at H+15mn)*
- *At the end of the game, the diary (the enigma mentioned earlier) recounted that she was wearing little support, despite her history of phlebitis and her recent fracture.*

**Figure 3:**
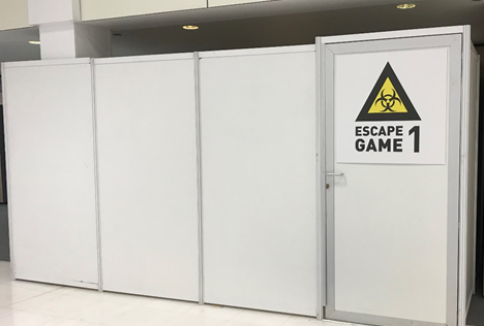
Exterior of the 12-m² escape room that was used for the emergency-medicine game in this study.

We made the game mobile by giving players access to the patient’s personal suitcase and belongings in a closed room (Figure 1). Our escape game is thus portable and can be easily transported on public transport by 1 or 2 people and with minimal equipment (trunk or suitcase) required. These items are then transported to a closed 12-m² space (Figure 3).

### Step 5: Create the Briefing and Debriefing Phases

#### Briefing

After listing the rules of the game (Multimedia Appendix 1), the facilitator introduces himself as an intern on the military base and hands over the video message from the local authorities, who then explains the mission to the team before the beginning of the game. The facilitator then leads the players into the room and the game begins.

#### Debriefing

Debriefing takes place immediately after the end of the game. After reviewing the diagnostic process, the facilitator first asks for the group’s opinion on their communication and then seeks to highlight with the group the strengths and weaknesses of their communication (self-assessment and heteroassessment as in the simulation [25]). To authenticate this, the facilitator also encourages players to talk about similar real-life experiences they may have had. The general setting of our debriefing is provided in Multimedia Appendix 2.

### Step 6: Testing the Game

#### Initial Testing

To be able to adjust gameplay, we tested the game completely with 2 teams of 5 players who work in health care. We advise testing with 2 different teams. We observed the interlocking nature of the different stages of our gaming and did not find any major issues. After the first run-through, we adjusted an enigma that was problematic with regard to readability (codes were hidden in jars containing elements of a recipe to be put back in order but the numbers were too difficult to read). This test allowed us to understand what the pivotal points of the game were; that is, code A must be solved before H+12 minutes, otherwise the game might not be completed in time. This allowed us to predict the times at which the facilitator should intervene. Thus, after adjustments, the second run-through was a success. In both cases, the evaluation from participants was positive.

#### Educational Evaluation

Educational evaluation using focus groups on the day and through questionnaires 3 month later will be the subject of a subsequent study.

## Discussion

### Overview

Our objectives were to define the creative stages needed to produce an educational escape game to help trainers who wish to develop a tool for learning team communication and the diagnostic approach. We believe that, to our knowledge, we have created the first ambulant, educational escape game that teaches staff (nurses, doctors, and paramedics), working in emergency medicine, to work as a team, and we have defined 6 steps for its creation.

The methodological setting that we have established can be used to create other games and is more detailed than that described in the literature [16-18] and was specifically created for the purpose of learning team communication and the diagnostic process in medical science.

Indeed, escape games could target a wide range of professions and can be used with most clinical cases. Our setting allows the creation of a multidisciplinary, mobile, and accessible game that promotes teamwork. Of note, our model also gives freedom to choose specific educational objectives. Educational escapes games are particularly adapted to the following objectives: the need for collegial decision-making and complex clinical situations involving several professions, and teamwork situations. All of these situations require excellent communication skills. Furthermore, in our opinion, any medical theme could be considered, even complex ethical ones.

While we were creating our game, we realized that student immersion is the key to educational success. It is also important that the game can take place at any time (present, past, or future) and at any location (hospital, outdoors, a house, or a public place). It could also be possible to invite our players to experience life aboard a spaceship.

The games we encountered in the literature [16-18,29,30] are not mobile like ours is; the mobility of our game is a real asset because it is easily transportable and can be played at any venue.

Nevertheless, in our opinion, a limitation of our proposed educational game is that it is not designed to develop technical medical skills but rather nontechnical skills such as communication. We could, however, well imagine puzzles that may involve technical skills (such as successful intubation to unlock a key).

The main objective of the escape game is to teach the players how to communicate with each other as a team and promote team cohesion [14,15]. Improved team cohesion is one of the most important benefits of collaborative learning methods (including games) that involve small training groups [31].

As with all educational games [4,15,18,19,29,30,32], we believe that our escape game could improve learning and the motivation to learn. Our game seems complementary to other games because it favors team cohesion, which is particularly important in emergency medicine. While there are other escape games in the literature, they only targeted medical students [18,30,32] or resident nurses/nursing students [19,29]; they did not aim to improve team dynamics.

Finally, since no roles are formally assigned, we work mainly on the evolution of the group and how it functions (eg, leadership is not assigned to an individual; rather, a leader often emerges in the group to help lead the group to a successful conclusion).

The escape game is similar to simulation-based training since it promotes team dynamics. Furthermore, they both involve a debriefing, which consolidates their educational impact. It should be noted that this debriefing exists in the escape game as part of the game and allows players to understand the puzzles they have experienced. The debriefing and its link with real-life situations provide authenticity to the situation and help consolidate the learning; this, in turn, results in successful gameplay.

However, differences can be observed in terms of educational emphasis that this game lays. The escape game emphasizes learning about the diagnostic process and communication skills without necessarily requiring undertaking technical and medical tasks. By contrast, simulation focuses on “real-life” technical and medical settings and is not an immersive game. Our game does not involve technical, medical, or paramedical activities; the situation is fictitious and aims at learning the diagnostic process and team communication. As we have seen, the learner is in a gaming situation rather than in a professional setting.

### Limitations

The main limitation of our paper is the setting and development of the educational escape game. It is indeed not possible to draw concrete conclusions on the effects of the game on students and the outcome or learning improvement, and we can only make assumptions.

### Conclusions

In our opinion, an escape game complements standard health training. It is particularly suitable for the field of emergency medicine, but our setting allows us to adapt it to any type of health profession or medical specialty. From an educational point of view, it may be a good tool for helping people in multidisciplinary fields (medical and paramedical teams) to learn how to work collaboratively and to communicate as a group. Above all, it is an innovative tool that complements medical simulation–based learning and thus consolidates traditional medical education. At present, a methodology for creating this type of educational game has not been proposed. Our team is currently evaluating the long-term educational benefits of our methodology and our game.

## Appendix

Multimedia Appendix 1 Addendum I: rules of the game.

Multimedia Appendix 2 Addendum II: debriefing plan.
