# Supplementary material for: A Didactic Escape Game for Emergency Medicine Aimed at Learning to Work as a Team and Making Diagnoses: Methodology for Game Development
Source: JMIR Serious Games. 2021 Aug 31;9(3):e27291. doi: 10.2196/27291 (PMC8441606; doi:10.2196/27291)
Supplement: Multimedia Appendix 1 [file games_v9i3e27291_app1.docx]

**Addendum I: rules of the game**

Move, lift, turn, flip, leaf through, examine, inspect, don't be afraid, search everywhere!

On the other hand, there is no need to force it, even if you are excited about the diagnostic process.

The only strength you need is that of your mind. So, if a padlock or an object doesn't open easily it's not meant to open. Objects covered with plaster are not meant to open. To avoid thinking about it too much, know that we will not respect time in this room, except the stopwatch of course.

There is nothing to be found beyond the space of the room, nothing is hidden in the walls or on your facilitator, but you can interact with him. Each padlock or code is used only once.

30 minutes, not a second more. Manage your time well and don't forget to communicate, just like in a complex clinical situation. Say aloud that you have found an object, solved a puzzle or opened a lock, and make sure that other team members have seen your progress.

And just between us, please don't write on our documents.

, GOOD LUCK!
