# Supplementary material for: A Didactic Escape Game for Emergency Medicine Aimed at Learning to Work as a Team and Making Diagnoses: Methodology for Game Development
Source: JMIR Serious Games. 2021 Aug 31;9(3):e27291. doi: 10.2196/27291 (PMC8441606; doi:10.2196/27291)
Supplement: Multimedia Appendix 2 [file games_v9i3e27291_app2.docx]

**Addendum II: Debriefing Plan.**

1. Clinical case debriefing: what really happened to the terrorist and the passengers? What were the elements to remember?

2. Group communication debriefing.

Debrief according to the attitude of the group. Highlight the positive points and explain the negative points.

-Begin with a caring attitude toward other participants.

-Determining the leader ship as early as possible.

-Clear and intelligible distribution of tasks.

-Communicate clearly an action, a discovery or a reflection.

-Repeat information that has not been gathered by the other participants.

-Do not just talk, but try to listen and share.

-Relate the clinical information of all patients to the findings of the search, in an ordered diagnostic process.

-Control the time allotted by designating an optional "time keeper".

-Impose pause times when the participants are respecting each other.

-Make regular summaries to reorganize the game plan.

-Allow time for conclusions and confirm responses to the problems posed in a collegial manner.
